# Residential History and Groundwater Modeling: Gallagher et al. Respond

**DOI:** 10.1289/ehp.1002444R

**Published:** 2010-09

**Authors:** Lisa G. Gallagher, Thomas F. Webster, Ann Aschengrau, Verónica M. Vieira

**Affiliations:** Boston University School of Public Health, Boston, Massachusetts, E-mail: vmv@bu.edu

We thank Schaider et al. for their interest in our research on possible environmental causes of breast cancer in upper Cape Cod, Massachusetts (Gallagher et al. 2010). Our study was prompted by an earlier spatial analysis that revealed a geographic overlap between groundwater plumes in upper Cape Cod and an area of increased breast cancer risk. These plumes indicated areas of concern around landfills and wastewater facilities, large point sources of contaminants to groundwater, as shown in Figure 1 of our article (Gallagher et al. 2010). We determined that among these plumes, the plume associated with the Barnstable Wastewater Pollution Control Facility (BWPCF) was the only point source with the potential to impact the drinking water of our study population. The BWPCF treats both residential and commercial waste from a broad geographic area.

In our study (Gallagher et al. 2010), we applied an extensive groundwater model to evaluate historic conditions and determined that effluent from the BWPCF could have reached public drinking water wells as early as 1966. Taking into account residential histories and drinking water source (public, private, and bottled water) among cases and controls, we found an association between Barnstable Water Company (BWC) drinking water impacted by the BWPCF plume and breast cancer that increased with longer latency and greater exposure duration. As Schaider et al. point out, drinking water contamination by private septic systems is ubiquitous in this area. However, because this source of pollution likely affects cases and controls across the entire study area in a similar manner and because the results of a prior study on this topic did not find an association (Brody et al. 2006), this unmeasured source of pollution should not confound the results of our analysis. Nevertheless, we do acknowledge in our article that there may be residual confounding by other unmeasured sources of environmental contamination, including the Barnstable Airport. We agree with Schaider et al. that these exposures may also contribute to the risk of breast cancer, although the earliest data we are aware of show no appreciable levels of volatile halogenated compounds in 1984 BWC water samples (Janik 1987). With only limited historical data available, we cannot be sure of the exact timing and geographic distribution of these other exposures. However, a very tight correlation would be necessary for these confounders to account for the observed associations with the BWPCF plume, and other exposures would have to date back to 1966.

Fortunately, groundwater sources of drinking water in this area are subject to more protections today, and we agree that ongoing monitoring of known and emerging contaminants is important to maintain water quality.
